# Intranasal pH and Olfactory Function Among Healthy Adults

**DOI:** 10.1002/lary.32247

**Published:** 2025-05-10

**Authors:** Anna Kristina Hernandez, Sero Biguerdi, Karoline Lantzsch, Katharina Schindowski, Nadine Gunder, Rumi Sekine, Eri Mori, Thomas Hummel

**Affiliations:** ^1^ Smell and Taste Clinic, Department of Otorhinolaryngology, Faculty of Medicine Carl Gustav Carus TU Dresden Dresden Germany; ^2^ Department of Otolaryngology—Head and Neck Surgery, Philippine General Hospital University of the Philippines—Manila Manila Philippines; ^3^ Department of Otolaryngology—Head and Neck Surgery Asian Hospital and Medical Center Muntinlupa Philippines; ^4^ Institute for Applied Biotechnology Biberach University of Applied Science Biberach an der Riss Germany; ^5^ Department of Otorhinolaryngology Jikei University School of Medicine Tokyo Japan; ^6^ Department of Otorhinolaryngology St. Luke's International Hospital Tokyo Japan

**Keywords:** mucus, nasal, olfaction, pH, smell

## Abstract

**Objectives:**

To determine whether the site of measurement (over respiratory mucosa (RM) or within the olfactory cleft [OC]), olfactory testing, or repeated pH testing affects pH measurements and whether OC pH affects olfactory function.

**Methods:**

This cross‐sectional study included 62 healthy adults (38 women, median age: 24 years). They underwent the following measurements: intranasal pH (Restech Dx‐pH; Respiratory Technology Corp., Houston, USA) and olfaction (“Sniffin' Sticks” Odor Identification Test [OdorId]; Burghart, Holm, DE). Participants were divided based on the following: sites of testing (RM [*n* = 27] vs. OC [*n* = 35]); order of testing (olfactory testing [OdorId] between two pH tests [pH‐OdorId‐pH, *n* = 17] versus two pH tests separated by a 20‐min interval followed by olfactory testing [pH‐pH‐OdorId; *n* = 18]). Comparisons between the first and second pH tests were also performed.

**Results:**

OC pH measurements were significantly lower than RM pH. Repeated pH testing resulted in subsequently more alkaline measurements, while olfactory testing had no effect. Odor identification scores correlated with OC pH but not with RM pH. In an exploratory linear regression model, OC pH predicted 18% of the variance in OdorId scores and was a significant negative predictor of OdorId performance (as pH increases, OdorId decreases).

**Conclusion:**

The OC pH is more acidic compared to that over the RM. Increased acidity in the OC was related to better olfactory function, warranting further investigation in olfactory dysfunction.

**Level of Evidence:**

NA.

## Graphical abstract

In this cross‐sectional study of 62 adults, olfactory cleft (OC) pH was lower than that over the respiratory mucosa (RM), with repeated testing resulting in more alkaline pH and olfactory testing having no effect. OC pH, but not RM pH, was negatively correlated with odor identification scores and predicted 18% of the variance in these scores. OC pH is different from RM pH, and a more acidic OC pH relates to better olfactory function.

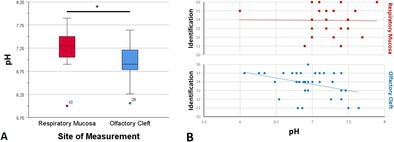

## 1 Introduction

The pH of a fluid or tissue is determined based on the concentration of hydrogen ions (H^+^) it contains [1]. Intranasal pH in healthy individuals has been reported to range between 5.3 and 7 and vary towards a more acidic trend with sufficient rest, adequate sleep, nasal vasoconstriction, and external heat in early studies [2, 3, 4, 5]. An acidic intranasal pH of ≤ 6.5 was reported to limit the growth of bacteria in the nasal cavity [6, 7]. Other investigations, however, have supported that pH may be more alkaline [6], up to 7.2–8.3 in individuals with acute or allergic rhinitis [3, 5] and may shift back to acidity as the condition improves [2]. A more alkaline pH has also been observed in men when compared to women [5], during the onset of menstruation in women [8] and after exposure to cold air [2]. In addition, a recent study reports that intranasal pH remains constant throughout the day and is not affected by daily activities such as eating, drinking, or sleeping [9] and is likewise unaffected by smoking, anatomical variations or age [5]. pH is also believed to influence various physiologic processes (e.g., perireceptor events [e.g., enzymatic activity and odorant metabolism]) [1, 10].

Clinical measurement of intranasal pH was previously limited by the lack of appropriate devices, as such equipment was primarily designed for gastroenterologists to measure esophageal pH [5, 11]. In recent years, there has been a noticeable rise in studies on intranasal pH, perhaps partly driven by the growing portability and accessibility of pH measurement devices. Notably, a previous study used a device similar to the one used in the present study and from the same manufacturer to investigate the effects of bacterial colonization in the sinonasal mucosa on intranasal pH [12]. There is a clear need to build on the limited research available by conducting more studies on intranasal pH and testing the reliability of these devices for use in the nasal cavity.

The current design of in situ pH measuring devices involves probe contact not only with the nasal mucus but also with the nasal mucosa. Ultimately, the recorded intranasal pH reading may be influenced by the H^+^ concentration of both the mucus and the mucosa. However, the nasal mucosa is generally believed to have a more stable pH compared to the nasal mucus, which may be affected by temperature, osmotic pressure, and evaporation among others [13]. Many of the existing literature on the topic refer to their in situ pH measurements as representing nasal mucus pH, but it is important to acknowledge that these pH readings may reflect contributions from both the mucus and the underlying mucosa. Hence, for the purposes of this study, pH measurements are discussed with respect to their specific intranasal locations (within the olfactory cleft [OC] or over the respiratory mucosa [RM]), in recognition of the potential contributions from both mucus and mucosa.

Although nasal mucus has been hypothesized to affect olfactory function through several mechanisms (maintaining tissue integrity of the olfactory epithelium (OE), protecting the OE from drying out, and providing an environment that is suitable for olfactory transduction [14, 15, 16, 17]), investigations that include nasal mucus pH and olfactory assessment are limited. Although there are some studies that investigate the olfactory microenvironment, specifically the presence of certain cytokines [18, 19], enzymes [20] and other proteins [21], these studies usually do not include functional olfactory testing [22]. To the best of the authors' knowledge, only one study explored intranasal pH (at the area of the inferior turbinate and the posterior of the maxillary spine) among patients with Parkinson's disease, a population that is associated with olfactory dysfunction [23]. Although only 27 patients were included and psychophysical olfactory testing was not performed, the mean intranasal pH was found to be significantly more alkaline in patients Parkinson's patients.

This study aims to determine: (1) if there are differences in pH measurements in various sites of the nasal cavity (comparing measurements over the respiratory mucosa versus within the olfactory cleft); (2) whether olfactory testing or repeated pH testing affects OC pH measurements; and (3) whether OC pH has an effect on olfactory function.

## 2 Materials and Methods

This prospective, cross‐sectional study included subjectively healthy adults (≥ 18 years) without any nasal complaints who were tested at the Smell and Taste Clinic, University Hospital Dresden, from March 2023 to September 2024. Individuals with nasal symptoms or a history of viral infection in the last four (4) weeks were excluded. In addition, one participant with incomplete data was excluded from the analyses. The study was approved by the local ethics committee (application number BO‐EK‐239052022).

All participants provided written informed consent and underwent quasi‐randomization into two groupings: first, those who had pH measurement over the RM versus those who had pH measures within the OC; second, the latter group of OC pH participants were further divided into those who had olfactory testing (OdorId) between two pH measurements (pH‐OdorId‐pH) versus those who had a 20‐min break between two pH measurements followed by olfactory testing (pH‐pH‐OdorId). A systematic medical history was taken from all participants (including questions on age, gender, nasal symptoms, olfactory function, and smoking history).

## 3 Outcomes

### 3.1 Intranasal pH

**FIGURE 1:**
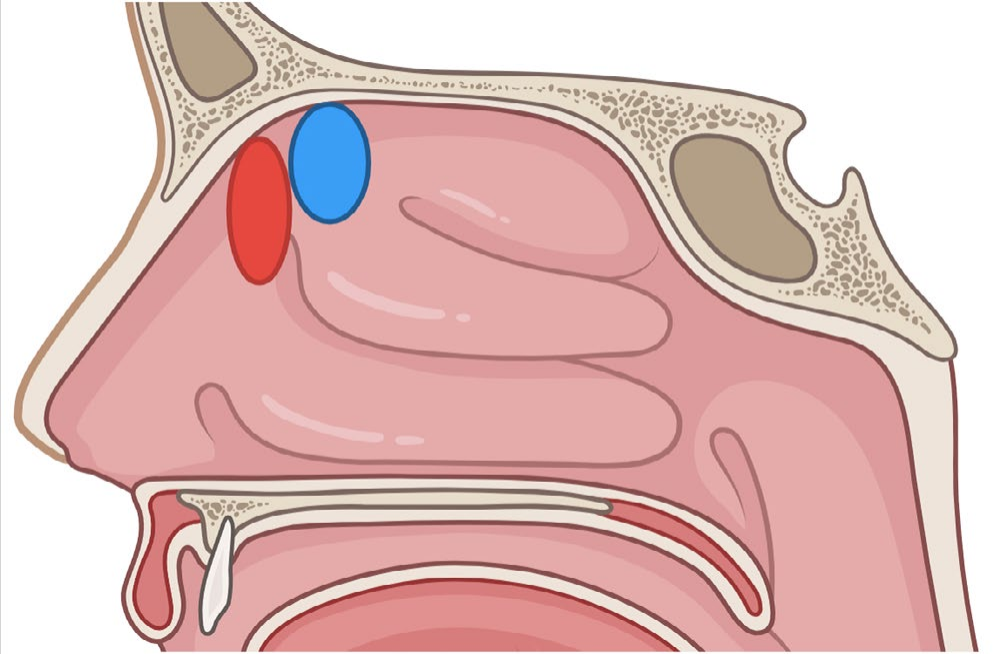
Anatomic location of pH probe placement. Red: Respiratory mucosa; blue: Olfactory cleft. Figure created in BioRender [24].

Intranasal pH was measured using the Restech Dx‐pH device (Respiratory Technology Corp., Houston, Texas, USA) using Dx‐pH Probes placed under endoscopic guidance by an otorhinolaryngologist over the RM (Adult sized Dx‐201 Probes placed anterior to the axilla of the middle turbinate [MT]) and within the OC (Pediatric sized Primus Probes placed between the medial surface of the MT and the nasal septum, but posterior to the most anterior surface of the MT; Figure 1). This pH device only requires hydration in a solution provided by the same manufacturer (Dx‐020H, Hydration vials) for 300 s or less. The pH value after 5 s of stable readings was recorded. Intranasal pH values were taken once for the RM group and twice for the OC group. The two OC pH measurements were separated by olfactory testing or a 20‐min break. Intranasal topical decongestants or anesthetics were not used during nasal endoscopy or endoscopic pH probe placement. Participants were advised to breathe normally through the nasal cavity.

### 3.2 Olfactory Test

Olfactory tests were performed using the “Sniffin' Sticks” Odor Identification Test (OdorId, Burghart Messtechnik, Holm, Germany) [25, 26]. The OdorId is a validated 16‐item suprathreshold test that uses devices similar to felt tip pens filled with commonly known odors. These pens were presented approximately 2 cm in front of participants' nostrils and they were asked to identify the odor from a selection of 4 descriptors. The score was based on the total number of correct answers, with a maximum score of 16.

### 3.3 Statistical Analysis

The sample size was determined using G*Power [27] (Version 3.1.9.7, https://www.psychologie.hhu.de/arbeitsgruppen/allgemeine‐psychologie‐und‐arbeitspsychologie/gpower; Germany). Effect sizes ranged from 0.44 [12] to 2.81 [23] in earlier studies. The minimum total sample size for an independent‐samples *t*‐test, with an effect size of 0.9, an alpha level of 0.05, and power of 0.9, was 54.

Data were analyzed using IBM SPSS Statistics (Version 29.0.2.0; IBM Corp., Armonk, NY, USA). Means and standard deviations (for normally distributed data) or medians and interquartile ranges (IQR) (for non‐normally distributed data) were determined for continuous variables, while frequencies and percentages were used for categorical variables. Independent sample t‐test was used to determine baseline group differences in age. Chi‐Square test was used to determine baseline group differences in sex and smoking history. Repeated measures analysis of variance (ANOVA) was performed to analyze differences between the first and second OC pH measurements. Pearson's *r* was performed to assess the correlations between OdorId and RM or OC pH, as well as the correlations between the first and second OC pH measurements. A linear regression, with OdorId as the dependent variable and OC pH as a predictor entered using the standard method, was performed to assess for possible predictors of OdorId. A *p* value < 0.05 was regarded as significant.

## 4 Results

**FIGURE 2:**
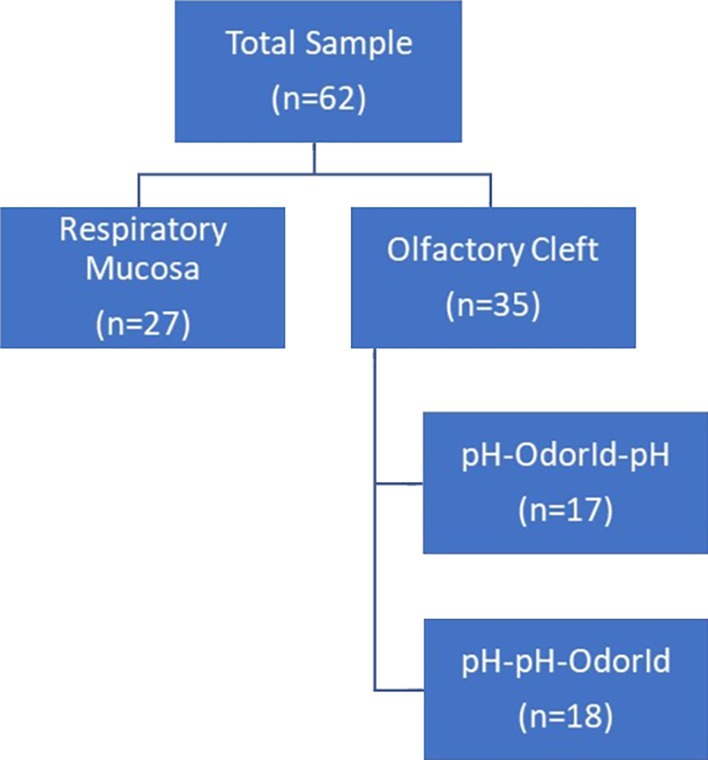
Sample and group distribution diagram. OdorId: “Sniffin' Sticks” odor identification test.

Sixty‐two participants were included in the study (38 women, 24 men; Age: Median[IQR] = 24[22.75–26] years). The participants were divided into two groups: Respiratory Mucosa (*n* = 27) and OC (*n* = 35), and the OC group was further divided based on the order of testing: pH‐OdorId‐pH (*n* = 17) and pH‐pH‐OdorId (*n* = 18, Figure 2). There were no significant differences between ages and OdorId scores between the two sets of groups.

**FIGURE 3:**
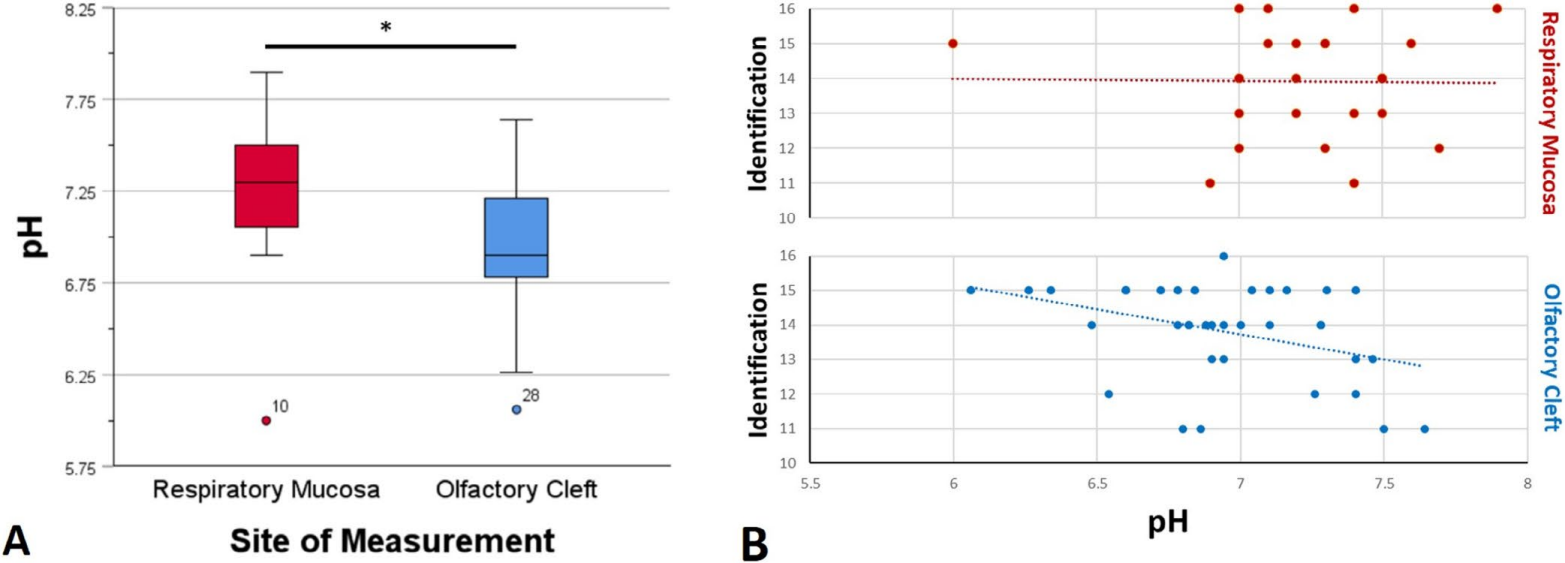
Intranasal pH Differences and Intranasal pH in relation to Odor Identification Test Scores over the Respiratory Mucosa and within the Olfactory Cleft. (A) Boxplot of intranasal pH measurements over the respiratory mucosa (RM) and in the olfactory cleft (OC). *p < 0.001; (B) Scatterplot of intranasal pH measurements over the RM and in the OC in relation to “Sniffin’ Sticks” Odor Identification test scores. OC pH was negatively correlated with Odor Identification Test scores (p = 0.01), while RM pH was not.

The mean OC pH (mean[SD] = 6.93[0.36]) was significantly lower compared to mean RM pH (mean[SD] = 7.26[0.35]; *t*_60_ = 3.51, *p* < 0.001; Figure 3A). There were no differences in OC pH based on the testing order, but the session had an effect, with the second pH measurement being higher than the first (*F*_1,33_ = 6.62, *p* = 0.02). The first and second OC pH measurements were also positively correlated (*r*_35_ = 0.58, *p* < 0.001).

OC pH (*r*_35_ = −0.43, *p* = 0.01), but not RM pH (*r*_27_ = −0.003, *p* = 0.99), was negatively correlated with OdorId (Figure 3B). In an exploratory linear regression model (*F*_1,33_ = 7.41, *p* = 0.10), OC pH predicted 18% of the variance in OdorId scores and was a significant negative predictor of OdorId performance (as OC pH decreases, OdorId increases; *B* = −1.65, SE B = 0.61, Beta = −0.43, *p* = 0.01).

As an exploratory analysis, we calculated the magnitude of pH changes between the 2nd and 1st OC pH measurements (Table 1). Since pH measurements are logarithmic, the magnitude of change is calculated using the formula: magnitude of change = 10^ΔpH^, where ΔpH refers to the difference between the 2nd and 1st OC pH. A 0.21 pH change corresponds to a decrease in H+ concentration by a factor of 1.62; while a 0.07 pH change corresponds to a decrease in H+ concentration by a factor of 1.17. The mean Δ OC pH was not significantly different between the groups based on an independent samples t‐test (*p* = 0.23). The Δ OC pH ranged from −0.54 (3.5‐fold increase in acidity) to 1.02 (10.5‐fold decrease in acidity).

**TABLE 1:** Mean differences between 2nd and 1st OC pH.

Group (order)	*N*	Mean Difference	Std. Deviation	Minimum	Maximum
pH‐OdorI‐pH	17	0.21	0.38	−0.54	1.02
pH‐pH‐OdorI	18	0.07	0.26	−0.52	0.44

*Note*: OdorI: “Sniffin’ Sticks” Odor Identification Test.

## 5 Discussion

This study highlights three main points: (1) OC pH is more acidic than that over the RM; (2) repeated OC pH testing resulted in subsequently more alkaline measurements while olfactory testing had minimal to no effect; and (3) OC pH (but not RM pH) was negatively correlated with OdorId scores and predicted 18% of the variance in these scores.

Differences in pH levels have been observed between locations such as the nasal cavity and sinuses, with the middle meatus being more acidic and the maxillary sinus more alkaline in one study. These variations have been associated with differences in bacterial flora [12]. Another study found that pH measurements taken from a more posterior electrode placement at the level of the inferior turbinate were more acidic compared to those from a more anterior placement. However, the electrodes used in the two locations differed (anterior: glass, posterior: antimony), which could have had an effect on the results [9]. In contrast, another study reported similar pH values between the inferior turbinate and nasal septum [5]. Unfortunately, the authors were unable to identify any earlier studies comparing intranasal pH in different locations that included measurements of the OC.

It has been suggested that breathing through the nose may cause fluctuations in intranasal pH, possibly due to the hydrolysis of carbon dioxide, which leads to the formation of carbonic acid and a more acidic pH [28]. In a study measuring intranasal pH over the inferior turbinate, pH levels fluctuated with breathing—between 7.5 during exhalation and as high as 9.0 during inhalation—and decreasing with breath‐holding [29]. In this study, however, both OC and RM pH measurements were taken in the superior nasal cavity, an area likely to receive less airflow compared to more inferior regions [30]. In addition, the device used in this study remained in the nasal cavity until a stable reading from each site was observed for at least 5 s. Although this pH device may not be as sensitive as the capillary electrodes used in the earlier study, each measurement duration lasted longer than one respiratory cycle, and the pH values are likely to average out. Despite this, a significant difference in pH was still observed, suggesting that factors other than breathing or airflow may play a larger role in pH variations.

Another possible factor that may affect pH differences between the two sites is that the nasal secretions overlying them originate from distinct sources: in the OC—from Bowman's glands and sustentacular cells; while in the RM—from the goblet cells [31, 32, 33, 34, 35, 36]. Although the majority of the nasal secretions probably come from submucosal glands that are present in both sites [37, 38, 39], Bowman's gland secretions are predominantly serous [35] and contain acidic, sulfated, or neutral mucopolysaccharides [40], ions, and water [41]; while goblet cell secretions predominantly contain mucin [35, 38, 41, 42, 43]. Mucins are large, negatively charged glycoproteins that bind to positive ions and can swell by absorbing water, depending on the ion content and pH [41, 44]. They are responsible for the gel‐like properties of mucus [45], which are essential for its protective, barrier, and host‐defense functions (i.e., airway innate immunity) [46, 47]. In the OC, a continuous flow of serous mucus facilitates the efficient removal of odorants, playing a crucial role in restoring sensitivity after odorant exposure [36]. The higher mucin content in the mucus over the RM and the ability of mucus to regulate H^+^ ion diffusion (as observed in the stomach [48]) may explain the more alkaline pH observed in this area compared to the more serious mucus in the OC.

Repeated pH testing has been shown to result in an increase of as much as 0.3 when measurements are taken within a period of 5–10 min [8]. Our study confirmed this finding even after a longer interval was enforced, possibly due to the need for endoscopic positioning of the pH probe twice (participants did not undergo the olfactory testing with the pH probe in place). Goblet cells respond to direct contact [41] and may be in line with the explanation proposed by Jackson & Turner [8], where repeated pH probe placement in the nasal cavity may trigger a reflex response, increasing nasal secretions (e.g., mucin from goblet cells) and resulting in a more alkaline pH reading.

The more acidic OC pH observed in this study aligns with previous findings, which show that the intranasal pH of healthy individuals is slightly more acidic when compared to the plasma pH of 7.4 [5, 49]. This mildly acidic environment may inhibit bacterial proliferation and could be optimal for the activity of certain enzymes, such as lysozyme, which functions in the disruption of bacterial cell walls [8, 10, 50]. Studies in mice have shown that the activity of specific enzymes, such as carboxyl esterase, was higher in the olfactory mucosa than in the respiratory mucosa [51, 52]. Furthermore, enzymes in both nasal mucus and saliva metabolize odorants, and these metabolites influence odor perception, contributing to the overall sensory experience [52, 53]. Given the critical role of odorant metabolism, it is plausible that the pH of the olfactory mucosa must remain slightly acidic to support these processes. Although exposure to odorants alone did not significantly alter intranasal pH levels, the slight acidic environment could still be important for optimal enzymatic activity and may account for the variance in odor identification scores that was attributed to OC pH. In addition, earlier models of perireceptor events related to olfactory transduction have highlighted the importance of free diffusion of odorant molecules through the mucus to the odorant receptor sites. Parasympathetic stimulation and local irritation of the nasal mucosa may result in watery [54] and mucoid [17] rhinorrhea, respectively. This, in turn, may result in increased viscosity of nasal secretions and increased total diffusion time by as much as 3.8‐fold in watery rhinorrhea and 11‐fold in mucinous rhinorrhea [17]. While these explanations may be somewhat simplistic, considering the complex nature of odorant metabolism and transduction, as well as acid–base homeostasis in nasal secretions, these factors may contribute to the observed pH differences in these two sites.

Although our study provides initial data on the relationship of OC pH and olfactory function, the sample primarily consisted of younger, healthy, and normosmic individuals. More studies are needed across a broader sample (exploring intra‐individual variability), including older individuals or those with olfactory loss, also exploring possible effects of nasal airflow and breathing on intranasal pH. Future research should explore different pH measuring devices, incorporate other psychophysical olfactory tests (i.e., odor threshold and odor discrimination), control for mouth versus nasal breathing, and examine the effect of various intranasal drug therapies on pH levels as an additional parameter of therapeutic efficacy. Further investigation into the potential role of intranasal pH, particularly in the OC, is an exciting area of research. Understanding how intranasal pH may impact olfactory function could provide valuable insights and guide the search for new therapeutic approaches for olfactory loss.

## 6 Conclusion

The pH in the olfactory cleft is more acidic than that in the respiratory mucosa, likely influenced by the differences in the composition of nasal mucus at these two sites (i.e., higher mucin content in the RM, resulting in more alkaline pH; and enzyme activity in the OC that requires a slightly acidic pH). While olfactory testing had limited to no effect on OC pH levels, repeated pH testing resulted in subsequently more alkaline pH values, potentially due to stimulation from the pH probe. OC pH, but not RM pH, was correlated with Odor Identification scores and was a significant negative predictor of olfactory performance. This suggests that the pH of the OC may significantly influence olfactory function, warranting further investigation.

## Conflicts of Interest

Since 2022 T.H. collaborated with the following companies: Sony, Tokyo, Japan; Smell and Taste Lab, Geneva, Switzerland; Takasago, Paris, France; Cyrano, Delray Beach, FL, USA; Cynexo, Trieste, Italy; Sentosphere, Paris, France; NOAR, Sao Paulo, Brazil; Baia Foods, Madrid, Spain; Burghart, Holm, Germany. The remaining authors declare no conflicts of interest.

## Data Availability

The data that support the findings of this study are available from the corresponding author upon reasonable request.
